# Association of Genetic Variant FVIII Gene and Factor VIII: A Pilot Study Among Hemophilia A Female Relatives in Saudi Arabia

**DOI:** 10.7759/cureus.42038

**Published:** 2023-07-17

**Authors:** Abdullah T Almohammadi, Osman Radhwi, Hatem AlAhwal, Ahmed Barefah, Salem Bahashwan, Ibraheem M Ashankyty, Majed Almashjari, Rawan Ayaz, Adel Al-Marzouki, Galila F Zaher, Hend Hussain, Abeer A Samman, Abeer Zakariyah

**Affiliations:** 1 Hematology, King Abdulaziz University, Faculty of Medicine, Jeddah, SAU; 2 Hematology Research Unit, King Fahd Medical Research Center, Jeddah, SAU; 3 Medical Laboratory Sciences, King Abdulaziz University, Faculty of Applied Medical Sciences, Jeddah, SAU; 4 Hematology, King Abdulaziz University, Jeddah, SAU; 5 Hematology Research Unit, King Fahad Medical Research Center, Jeddah, SAU; 6 Internal Medicine, King Abdulaziz University, Faculty of Medicine, Jeddah, SAU; 7 Genetics, University of Jeddah, Jeddah, SAU

**Keywords:** aptt, factor viii, exon 14, female carriers, hemophilia

## Abstract

Hemophilia A (HA) is an X-linked recessive disorder that results from mutations in the factor VIII gene (FVIII). Most affected patients are males due to the inheritance of mutations in the FVIII gene from their mothers. Females are mostly found to be carriers unless they inherited the mutation from both parents. Obligate carriers of HA are mothers whose sons are affected with HA, or daughters who inherit the mutation from their affected fathers. A possible carrier of HA could be any female who has one or more affected relatives with HA in her family. Hemophilia A carriers (HACs) could present with similar symptoms to affected patients, including low factor VIII level, and risk of bleeding especially after surgical procedures or postpartum hemorrhage.

Objectives: Assessing the phenotype of possible HAC and its association with genetic variants in the FVIII gene for better screening methods for HAC.

Methods: From the period between 25 June and 25 October 2021, the study was conducted at King Abdulaziz University Hospital in Jeddah, Saudi Arabia. We recruited seven mothers whose sons were affected with HA, and 18 possible HAC who are relatives to sever affected patients with HA. All 25 candidates were assessed for the FVIII level, activated partial thromboplastin time (APTT), and bleeding risk and sequenced a part of Exon14 in their FVIII gene.

Results: Twenty-five percent of the participants show a low level of FVIII, however, none of them have prolonged bleeding nor suffer from bleeding tendency. We also identified two missense variants in six of the candidates, but the clinical significance of these variants has not been determined previously.

Conclusion: This pilot study is the first to explore the phenotype of several HAC in Saudi Arabia. A larger scale study with more HA patients and their female relatives is needed to understand the correlation between phenotype and genotype for better screening for HAC.

## Introduction

Hemophilia A (HA) is a genetic bleeding disorder that is inherited in a recessive X-linked pattern. HA is caused by heterogeneous mutations in the factor VIII gene in the X chromosome, which is responsible for the production of coagulation factor VIII (FVIII) protein [1]. Recurrent spontaneous hemorrhage or aberrant bleeding following an injury or surgical intervention are the defining characteristics of severe HA (< 1% of normal FVIII activity), which has the potential to be fatal if it is not diagnosed promptly [1,2]. Patients exhibit severe HA when FVIII activity is <1%, moderate HA when FVIII activity is 1-5%, and mild HA when exhibiting 6-40% activity, otherwise considered normal. Typically, HA is diagnosed by clinical examinations and coagulation factor assay [1,3].

Hemophilia A carriers (HACs) are females who inherit a pathological variant in the FVIII gene. Obligate carriers of HA are mothers whose sons are affected with HA, or daughters who inherit the mutation from their affected fathers. A possible carrier of HA could be any female who has one or more affected relatives with HA in her family [2,4,5]. Symptoms of bleeding can appear obscurely in HACs and arise in situations such as surgery, teeth extraction, tonsillectomy, and primary postpartum hemorrhage [3,5]. Suspected HACs must have their bleeding risk evaluated to provide an early diagnosis and support medical care [2].

The activity of FVIII is influenced by age, blood type, menstrual cycle, pregnancy, and other physiological factors in HACs [5,6]. Hemophilia carriers may be asymptomatic, but they pose a risk of passing the pathogenic variant to their offspring [7]. Consequently, some studies have suggested that molecular genetic analysis can be undertaken as soon as feasible to identify accurate variations of the FVIII gene in HACs and thereby avert major difficulties in HACs patients [3,6,8]. Early identification of HACs will give them the chance to be involved in treatment programs which will contribute to an increase in life expectancy and a decrease in the death rate [9,10]. The implementation of proper molecular genetic analysis techniques will provide numerous benefits to healthcare, therapy, and the development of preventive health programs to reduce the incidence of hemophilia-related morbidity and mortality [11,12].

Female HACs are anticipated to have a plasma concentration of FVIII equal to half the level found in normal individuals. However, in HACs, an extended range in coagulation factor levels is observed, from extremely low, like affected males with HA, in addition to the upper limit of normal [10]. To help physicians manage and care for HACs, it is vital to evaluate the bleeding tendency, such as establishing a prophylactic intervention in HACs at risk for bleeding [11].

Female HAC-related information is a novel notion. There has not yet been a genetic screening or genetic counseling investigation to determine the status of the HACs in Saudi Arabia. In HA patients, bleeding phenotype is influenced by a variety of factors including FVIII gene mutation which is categorized as null or non-null mutation [13]. A null mutation is consistent with the most common mutation which accounts for 45% of all cases. Moreover, it is also considered the one responsible for the severe bleeding phenotype in HA patients [12].

Integration of carrier testing and prenatal diagnosis as a part of comprehensive hemophilia care have become essential in many countries. Most of the carriers are interested in genetic testing if they are aware of the possibility [14]. While extensive information on genotype and phenotype in men with hemophilia is available, only a few studies have focused on genotype and bleeding in HACs [15].

This study aims to explore the phenotype of 25 possible HACs who are relatives of nine severe HA patients. Phenotype assessment includes measuring their FVIII level, activated partial thromboplastin time (APTT), and bleeding tendency. Moreover, the study aimed to detect possible single nucleotide polymorphism (SNPs) in the FVIII gene (exon 14) in the HACs.

## Materials and methods

This is a cross-sectional study that was conducted from the period between 25 June and 25 October 2021, among the female relatives of hemophilia patients in Jeddah, Saudi Arabia. Twenty-five females relatives of nine HA male patients who were already diagnosed and are followed in periodically manner in the Hematology Clinic at King Abdulaziz University Hospital (KAUH) were selected to participate in this study. The female’s relation to HA patients is shown in Table 1. Three patients from family numbers 2, 3, and 4 brought their mothers. Four patients from families 5, 6, 8, and 9 brought their mothers and sisters. A patient from family 7 brought his four sisters, and lastly, a patient from family 1 brought his six sisters and three nieces. Mothers are considered the obligate carriers. The rest of the participants including sisters and nieces are considered possible HACs. All 25 participants were above the age of 18 years. Approval (Reference No. 682-20) was taken from the institutional review board and written informed consent was obtained from all the participants.

**Table 1:** Representation of the 25 candidates in our study (females relatives to the nine severe HA patients). The table shows the family number (total is nine), candidate number (total is 25), the FVIII levels, APTT for both the patients and their relatives, and their relation to patients. It also shows the Cumulative Score which represents the bleeding tendency. It also represents the identification of SNPs in some candidates. Based on our laboratory at King Abdulaziz University the normal APTT range is 25-35 seconds. FVIII: factor VIII, APTT: activated partial thromboplastin time test, SNPs: single nucleotide polymorphisms

Family #	Candidate #	Patient's FVIII Level (50-150%)	Patient's APTT (25-35 sec)	Relation to Patient	FVIII Level (50-150%)	Relative's APTT (25-35 sec)	Cumulative Score	SNPs
No. 1	1	0.2	97.8	Sister	32.9%	40.9	0	
	2	0.2	97.8	Sister	22.8%	40.2	0	rs781800942
	3	0.2	97.8	Sister	58.6%	37.3	0	rs782427638
	12	0.2	97.8	Niece	99.9%	33.7	0	
	13	0.2	97.8	Niece	57.3%	35.7	0	
	14	0.2	97.8	Sister	74.3%	41.9	0	
	15	0.2	97.8	Niece	113.1%	35.6	0	rs782201289-rs1345691509-rs781800942
	24	0.2	97.8	Sister	67.2%	37	0	
	25	0.2	97.8	Sister	132.1%	35.4	0	
No. 2	4	0.5	111.5	Mother	107.1%	31.2	2	rs782201289
No. 3	5	0.5	103	Mother	22.5%	38.9	0	rs782427638-rs781800942
No. 4	6	0.5	97	Mother	55.3%	31.5	0	rs782427638-rs781800942
No. 5	7	0.5	115.4	Sister	66.4%	27.9	0	
	8	0.5	115.4	Mother	46.8%	31.5	1	
	9	0.5	115.4	Sister	64.0%	31.3	0	
No. 6	10	0.3	106	Sister	82.5%	34.6	0	
	11	0.3	106	Mother	10.1%	28	0	rs782427638
No. 7	16	0.9	97	Sister	84.4%	32.6	2	
	17	0.9	97	Sister	77.1%	34.7	0	rs782427638-rs781800942
	18	0.9	97	Sister	56.5%	32.3	0	
	19	0.9	97	Sister	85.7%	28.6	1	
No. 8	20	0.1	115	Sister	61.7%	39.3	1	
	21	0.1	115	Mother	52.2%	36.8	0	rs782427638
No. 9	22	0.1	101	Sister	141.3%	33.3	2	
	23	0.1	101	Mother	43.0%	38	0	

For studying the coagulation profile in the candidates, a venous blood sample was collected in 3.2% buffered sodium citrate (blue top vacutainer tube). All participants were tested for FVIII level that was carried out on ACL TOP 550 coagulation analyzer (Werfen, Massachusetts, United States) and determined by performing an APTT. Participants' plasma samples were diluted and then added to FVIII deficient plasma. The correction of the clotting time of the deficient plasma is proportional to the correction (% activity) of FVIII in the patient’s plasma interpolated from a calibration curve. Testing of APTT and prothrombin time (PT) were carried out on the same machine ACL TOP 550. In the APTT test, the incubation of a plasma sample with an optimal quantity of phospholipid and buffer initiates the activation of the intrinsic coagulation pathway after incubation at 37°C for 5 minutes, calcium was then added to trigger the coagulation process and the time required for clot formation is measured. In the PT test, participant plasma was incubated at 37°C in the presence of calcium chloride prior to the addition of tissue thromboplastin (Recombi Plas Tin 2G) reagent to initiate the activation of the extrinsic coagulation pathway and finally clot formation. The test is timed from the addition of calcium chloride until the plasma clots.

Bleeding risk was assessed in all candidates by the International Society of Thrombosis and Homeostasis-Bleeding Assessment Tool (ISTH-BAT) questionnaire [16], which contains 14 questions about bleeding in different body sites including skin, oral cavity, gastrointestinal system, genitourinary system, muscle, joint, central nervous system, and as well bleeding response to challenging situations as tooth extraction, post-surgical procedures, and post-partum hemorrhage [16]. Each question contains scoring points from 0 to 4, depending on the bleeding amount and whether any intervention was taken to control bleeding. Patients were asked after the interview to go to the main laboratory department to withdraw blood which will be used to investigate for FVIII level, PTT in addition to genetic testing for mutations.

For deoxyribonucleic acid (DNA) extraction and mutation analysis blood samples were collected from each candidate in an ethylenediamine tetraacetic acid (EDTA) vacutainer and were used to extract whole genome DNA using the QIAamp DNA formalin-fixed, paraffin-embedded (FFPE) Tissue kit (Qiagen, Germantown Maryland, United States) (Cat. # 56404) by following the manufacturer's instructions. DNA purity and concentration were determined using NanoDrop™ 2000/2000c, Spectrophotometers (Thermo Scientific, Maryland, United States). Around 20-100 ng of DNA was used for each gradient polymerase chain reaction (PCR) run. We sequenced a specific region of the FVIII gene, which targets a part of Exon 14. One set of primers was used to amplify the region. The sequences of primers used for the amplification of the FVIII gene involved in this study are listed in Table 2. The PCR thermal program for detection is composed of 35 cycles of denaturation at 95°C for 30 secs, followed by annealing at 56°C for 30 secs, and an extension at 72°C for 1 min. Cycling was initiated with the first denaturation at 95°C for 5 min and concluded with a final extension at 72°C for 5 min. Gradient PCR products of 1440 bp were analyzed by 1.5% agarose gel electrophoresis. Sequencing was performed using a BigDye Terminator v3.1 cycle sequencing kit on an Applied Biosystems 3730xl DNA Analyzer (Life Technologies, Carlsbad, Canada). The sequence results were aligned with the gene bank using nucleotide BLAST (BLAST software at https://ncbi.nlm.nih.gov/blast).

**Table 2:** Primer used for amplification and sequencing

Exon No.	Forward Primer	Reverse Primer
Exon 14	5’-CCTTGGTTTGCACACAGAAC-3’	5’-ATCTTGAAGTACTGGAGCAT-3’

## Results

APTT and FVIII level indicated that there was a slight increase in APTT in nine candidates and FVIII level was reported to be less than 50% in only six candidates. Only four out of the six who have low FVIII of less than 50% had prolonged APTT (see Table 1).

In order to assess the bleeding risk for the candidates, we interviewed 25 candidates using the ISTH-BAT questionnaire. Each question contains scoring points from 0 to 4 depending on the amount of bleeding and if there was intervention had been taken to control bleeding. The scoring has been judged based on patient history given during the interview by the hematologist during the clinic visit with the participants. The normal range of total score is less than 6 for adult females. None of the 25 candidates were at risk of inherited bleeding disorder based on the bleeding score.

In order to explore for SNPs in a part of the FVIII gene we choose Exon 14 since it is the largest coding sequence which makes it prone to mutations [17,18]. Genomic DNA samples from the 25 candidates were collected for DNA sequencing. After performing PCR using the primers indicated in Table 2, DNA sequencing results were assembled to MN_000132.4 using BLAST-NCBI and Geneious software. We found four SNPs in nine of the candidates as shown in Table 3. We used https://www.ncbi.nlm.nih.gov/snp/?term= to investigate the significance of the SNPs identified. Two of the SNPs were missense variants. The first SNP (found in one candidate) is rs1345691509, which results in changing the protein sequence at amino acid 836 from Serin to Proline (p.Ser836Pro). The other SNP (found in five candidates) is rs781800942, which results in changing the protein sequence at position 1113 from Methionine to Threonine (p.Met1113Thr). The other two SNPs were synonymous and did not change the protein sequence and were found in eight of the candidates.

**Table 3:** Shows the SNPs identified in several candidates in our study and the effect of the SNPs on the amino acid sequence. SNP: single nucleotide polymorphisms, cDNA: copy deoxyribonucleic acid

SNP ID	Amino Acid Position	Codon Position	Protein Name	Function	cDNA Name	Candidate #
rs1345691509	836	1	p.Ser836Pro	missense	c.2506T>C	15
rs781800942	1113	2	p.Met1113Thr	missense	c.3338T>C	2, 5, 6, 15 and 17
rs782201289	776	3	p.Asn776Asn	synonymous	c.2328T>C	4, 11
rs782427638	813	1	p.Leu813Leu	synonymous	c.2437T>C	3, 5, 6, 11, 17 and 21

## Discussion

HA is caused by hundreds of different mutations and is manifested in clinical conditions of varying severity [14,17,19]. Even in the same mutation type, phenotypes may manifest differently [20]. The bleeding tendency in carriers is often neglected by both the carriers and their physicians as the correlation between bleeding tendency secondary to hemophilia and the female gender is rarely established [9]. Female HACs can present with FVIII levels within the normal range but might also report a considerable tendency to bleed episodes [21].

This is the first study in Saudi Arabia that focused on possible and obligate HACs who were relatives of nine severe HA patients. In this current cross-sectional study, we characterized 25 candidates in terms of their FVIII activity, APTT, and their bleeding risk. We also performed sequencing in a part of Exon 14 of the FVIII gene.

It was recently reported in many studies that low FVIII levels are consistently associated with clinically significant bleeding and correlate well with skewed X chromosome inactivation (XCI) [4,8]. Most interestingly, bleeding tendency is also observed in some HACs with normal FVIII levels and requires further investigation. Well-controlled studies investigating peripartum and periprocedural FVIII levels and adequate hemostatic treatment are necessary to inform management guidelines [22].

Female HACs have an increased bleeding phenotype as characterized by a growing number of studies [4,23-26]. Although in the current study, 24% of the candidates have FVIII below 50% and none of the candidates suffered from bleeding tendencies according to the bleeding tendency questionnaire. According to previous studies, HACs have bleeding with minor injuries, bleeding following medical interventions, heavy menstrual bleeding, muscle hematomas, and joint bleeding, ranging from 8% to 16% [24,25,27].

Direct mutation analysis for genetic diagnosis of hemophilia families is still not widely practiced in most developing countries including Saudi Arabia. Restriction fragment length polymorphism (RFLP) analysis using the polymorphic markers of the FVIII gene is more prevalent in developing countries for the genetic diagnosis of hemophilia families [7,28].

Our study includes 25 females who are relatives of nine HA patients. The mothers of some HA patients are obligate carriers. However, the other females’ relatives are possible HACs. A known obligate carrier female can be used for initial mutation identification [29]. Several studies have discovered many types of mutations which have been associated with HA in the FVIII gene, especially in Exon 14 [17,19,30]. Therefore, we search for possible SNPs in a part of Exon 14 in our candidates. Our search for SNPs has identified two missenses that result in changing the amino acid sequence as shown in Table 3. We searched for the clinical significance of these SNPs using https://f8-db.eahad.org/ and https://www.cdc.gov/ncbddd/hemophilia/champs.html databases, which include the mutations that have been reported or published from HA patients or carriers. We did not find any of the SNPs reported before. These two SNPs have not been reported nor published and were found in five of the candidates tested including two mothers, two sisters, and one niece. None of these five candidates have bleeding risk. We found two of the candidates, number 2 is a sister and number 5 is a mother, who have these SNPs and had low FVIII levels, but normal APTT. On the other hand, the other three candidates who had these SNPs showed normal FVIII levels and APTT. The variability in phenotype among the HACs is poorly understood [2]. Also, we cannot rule out the possibility that there are more SNPs on the other region of the FVIII gene since we just sequenced a part of Exon 14. Variability in FVIII among HAC is poorly understood. Therefore, more studies are needed to find out whether there is an association between the type of FVIII gene variant and plasma FVIII level in HACs.

It is worth noting that our study has a few limitations that should be taken into consideration, including the small number of participants. Such a shortcoming could be solved by recruiting more HACs in future studies. Moreover, and due to financial restriction, our report could have missed reporting other variants/SNPs in HAC as we focused on sequencing the hotspot area of Exon 14 in the FVIII gene that has been reported to harbor numerous mutations. Further studies will include the sequencing of the whole FVIII gene to detect point mutation and using the multiplex ligation‐dependent probe amplification method (MLPA) to detect deletions or duplications in the FVIII gene. Additionally, screening for novel pathogenic variants in the FVIII gene should be performed first on HA patients and then compare with the obligate carriers in their family such as their mothers. The identification of pathogenic variants in HA patients will allow for direct screening for their female relatives. It could also help in evaluating the validity of the prenatal genetic diagnosis of fetuses.

## Conclusions

In conclusion, the aim of our pilot study is to evaluate the feasibility of an approach that might be helpful in screening for HACs. This is the first study from Saudi Arabia that investigate the phenotype of possible HACs and detect SNPs in their FVIII gene and could help understand the correlation between genotype and phenotype as well as help screening for HACs in the future. Despite that 24% of the candidates have FVIII below 50%, most of them show normal APTT and none of the candidates have a bleeding tendency. We found two SNPs that have not been reported previously in the FVIII gene. Despite our effort in understanding the association between phenotype and genotype of possible HAC, our study here cannot conclude this association.
